# Characteristics and biomarkers associated with mortality in COVID-19 patients presenting to the emergency department

**DOI:** 10.1017/S0950268824000633

**Published:** 2024-04-19

**Authors:** Joon Oh Park, Hyun Kyu Cho, Cheon Hoo Jeon, Si-Ho Kim, Ik Hyun Park, Kwang Min Kim, Junho Lee, Yu Mi Wi

**Affiliations:** 1 Department of Internal Medicine, Samsung Changwon Hospital, Sungkyunkwan University School of Medicine, Changwon, Republic of Korea; 2 Division of Pulmonary and Critical Care Medicine, Department of Medicine, Samsung Changwon Hospital, Sungkyunkwan University School of Medicine, Changwon, Republic of Korea; 3 Division of Infectious Diseases, Department of Medicine, Samsung Changwon Hospital, Sungkyunkwan University School of Medicine, Changwon, Republic of Korea; 4 Division of Cardiology, Department of Medicine, Samsung Changwon Hospital, Sungkyunkwan University School of Medicine, Changwon, Republic of Korea; 5 Division of Gastroenterology, Department of Medicine, Samsung Changwon Hospital, Sungkyunkwan University School of Medicine, Changwon, Republic of Korea; 6 Department of Emergency Medicine, Samsung Changwon Hospital, Sungkyunkwan University School of Medicine, Changwon, Republic of Korea

**Keywords:** biomarkers, COVID-19, emergency department, extrapulmonary, mortality

## Abstract

This study aimed to investigate the diverse clinical manifestations and simple early biomarkers predicting mortality of COVID-19 patients admitted to the emergency department (ED). A total of 710 patients with COVID-19 were enrolled from 6,896 patients presenting to the ED between January 2022 and March 2022. During the study period, a total of 478 patients tested positive for COVID-19, among whom 222 (46.4%) presented with extrapulmonary manifestations of COVID-19; 49 (10.3%) patients displayed gastrointestinal manifestations, followed by neurological (n = 41; 8.6%) and cardiac manifestations (n = 31; 6.5%). In total, 54 (11.3%) patients died. A Cox proportional hazards model revealed that old age, acute kidney injury at presentation, increased total leukocyte counts, low platelet counts, decreased albumin levels, and increased LDH levels were the independent predictors of mortality. The albumin levels exhibited the highest area under the curve in receiver operating characteristic analysis, with a value of 0.860 (95% confidence interval, 0.796–0.875). The study showed the diverse clinical presentations and simple-to-measure prognostic markers in COVID-19 patients presenting to the ED. Serum albumin levels can serve as a novel and simple early biomarker to identify COVID-19 patients at high risk of death.

## Introduction

Severe acute respiratory syndrome coronavirus 2 (SARS-CoV-2), the causative pathogen of coronavirus disease 2019 (COVID-19), leads to a broad range of clinical manifestations, from mild respiratory symptoms to multiple organ failure [1]. Although the most frequent clinical syndrome of SARS-CoV-2 leading to hospitalization is pneumonia associated with severe hypoxemia and acute respiratory distress syndrome, healthcare professionals have also observed numerous extrapulmonary manifestations of COVID-19 [1, 2]. Extrapulmonary COVID-19 encompasses various disorders such as cardiovascular, coagulation, endothelial, renal, gastrointestinal, hepatobiliary, endocrinological, and neurologic impairment [1, 2]. Therefore, these diverse clinical manifestations of COVID-19 pose a diagnostic challenge, impede early detection, and lead to spread especially in the emergency department (ED) setting [3].

Numerous biochemical markers, including cytokines, procalcitonin, cardiac markers, lactate, lactate dehydrogenase (LDH), C-reactive protein (CRP), D-dimer, aspartate aminotransferase (AST), neuron-specific enolase, neutrophil count, neutrophils-to-lymphocytes ratio, brain natriuretic peptide (BNP), and its N-terminal pro-hormone have been associated with the severity of COVID-19 [4, 5]. However, the limited availability of certain markers hinders their widespread clinical application. Moreover, most earlier analyses focused on general inpatient or intensive care cohorts, with a paucity of dedicated investigations on ED populations. Identification of prognostic factors in this vulnerable population is crucial for optimizing case management and allocation of healthcare resources, particularly in resource-limited settings.

Contemporary data characterizing the prevalence and spectrum of extrapulmonary manifestations among COVID-19 patients visiting ED remain scarce. Therefore, this study aimed to investigate clinical manifestations and predictors of mortality in patients with COVID-19 who presented to the ED. Additionally, we sought to assess the correlation of commonly measured biomarkers with mortality in adult patients affected by COVID-19. Findings from this analysis will delineate high-risk patient attributes to guide clinical decision-making and emergency care in COVID-19.

## Methods

### Study population and design

Data for this study were obtained from the COVID-19 Samsung Changwon Hospital Registry Database. We included patients aged >19 years with COVID-19 infection who were admitted to the ED of a 760-bed referral centre between January 1, 2022, and March 31, 2022. The age cut-off of 19 years was chosen to align with the definition of adult patients at our institution. Throughout the study period, all patients presenting to the ED with respiratory symptoms or fever were routinely tested for SARS-CoV-2 infection. Furthermore, all admitted patients, regardless of the presence or absence of respiratory symptoms or fever, were screened for SARS-CoV-2 infection. The infection control department regularly monitored the results of laboratory tests for active hospital surveillance and relevant case details were recorded. The medical records of patients with confirmed COVID-19 were retrospectively reviewed, including demographic information, underlying comorbidities, clinical manifestations at presentation, laboratory findings, vaccination status, date of vaccination, and date of confirmed diagnosis of COVID-19. Thirty-day mortality was recorded as an objective marker of the severity of the disease. This study was approved by the institutional review board (SCMC 2022-10-019). The need for informed consent was waived due to the retrospective nature of the study.

### Definition

A confirmed COVID-19 case was defined as a positive result in a reverse transcriptase-polymerase chain reaction assay for SARS-CoV-2 in nasopharyngeal or oropharyngeal swab specimens or sputum. The full vaccination group included patients 14 days after the second vaccination. Blood tests for complete blood cell count and blood chemistry were simultaneously performed at admission, and the initial laboratory values upon hospital admission were used for the analysis. Albumin were measured as part of the automated chemistry analysis using a Roche Modular D2400 system (Roche Diagnostics, Indianapolis, IN), and the reference range of our institution was 3.1–5.2 g/dl. Complete blood cell counts were obtained using a Sysmex XN-10 hematology analyser. The diagnosis of acute kidney injury (AKI) was made using KDIGO criteria as an increase in serum creatinine 1.5 times the baseline creatinine within the previous 7 days or an increase in serum creatinine by ≥0.3 mg/dl within 48 h. Additionally, a urine volume of ≤0.5 ml/kg/h for 6 h was considered a criterion for the diagnosis of AKI.

### Statistical analyses

Discrete data were presented as frequencies and percentages, while continuous variables were summarized as mean ± standard deviation or median and interquartile range. Before analysis, the normality of the data was assessed using the Kolmogorov–Smirnov normality test. Appropriate statistical tests were used to compare the characteristics of between-subgroup survivors and non-survivors. The χ2 test, Fisher’s exact test, two-sample *t*-test, or Mann–Whitney *U* test were used depending on the nature of the data. To identify predictors of mortality in COVID-19 patients, a Cox proportional hazards regression model was used to control the influence of confounding variables. In this analysis, variables with a *P* value <0.05 in the bivariate analysis were considered candidates for the multivariate analysis, and a backward stepwise variable elimination process was performed. To predict mortality, receiver operating characteristic (ROC) curves were plotted. The criteria used for variable selection in the ROC analyses were: 1) objective laboratory test able to be measured within 24 h of hospital admission and 2) multivariate association with 30-day mortality at *P* < 0.05 level. Pairwise comparisons of the area under the curve (AUC) were performed using the DeLong method with Bonferroni correction. The AUC, sensitivity, and specificity were calculated to evaluate the predictive performance. A *P* value <0.05 was considered statistically significant. All statistical analyses were performed with SPSS Statistics 23.0, for Windows (SPSS Inc., Chicago, IL, USA), while the ROC curve analysis was performed with MedCalc v. 22.002 (MedCalc Software, Mariakerke, Belgium).

## Results

### Prevalence of COVID-19 and extrapulmonary manifestations

Among the 6,896 patients who visited the ED during the study period, 710 patients (10.3%) tested positive for COVID-19. The 232 excluded patients were those under 19 years old. Of the 478 enrolled COVID-19 patients aged over 19 years, 222 (46.4%) presented with extrapulmonary manifestations, with gastrointestinal symptoms being the most common (10.3%), followed by neurological (8.6%) and cardiac (6.5%) manifestations. Overall, 11.3% (n = 54) of patients died during the study period (Table 1).

**Table 1.** Clinical characteristics of COVID-19 patients presenting to the emergency department

	Total (n = 478)	Non-survivor (n = 54)	Survivor (n = 424)	*P* value
Age (years), mean ± SD	58.1 ± 19.4	71.9 ± 12.6	56.4 ± 19.5	<0.001
Male sex	248 (51.9)	34 (63.0)	214 (50.5)	0.084
Diagnosis to ED presentation (days), median (IQR)	0 (0–11.0)	0 (0–3)	0 (0–12.8)	0.067
Full vaccination status	318 (66.5)	29 (53.7)	289 (68.2)	0.034
Underlying disease				
Diabetes	65 (13.6)	11 (20.4)	54 (12.7)	0.123
Chronic lung disease	15 (3.1)	5 (9.3)	10 (2.4)	0.019
Liver cirrhosis	12 (2.5)	4 (7.4)	8 (1.9)	0.036
Cancer	66 (13.8)	12 (22.2)	54 (12.7)	0.057
Chronic heart disease	54 (11.3)	8 (14.8)	46 (10.8)	0.386
Cerebrovascular disease	42 (8.8)	7 (13.0)	35 (8.3)	0.302
Chronic renal disease	56 (11.7)	12 (22.2)	44 (10.4)	0.011
Clinical manifestation				
Extrapulmonary manifestations	182 (38.1)	22 (40.7)	160 (37.7)	0.002
Neurologic manifestations	41 (8.6)	5 (9.3)	36 (8.5)	0.798
Gastrointestinal manifestations	49 (10.3)	2 (3.7)	47 (11.1)	0.092
General weakness causing trauma	28 (5.9)	2 (3.7)	26 (6.1)	0.757
Cardiac manifestations	31 (6.5)	4 (7.4)	27 (6.4)	0.798
Acute kidney injury	16 (3.3)	6 (11.1)	10 (2.4)	0.005
Psychiatric disorder	7 (1.5)	2 (3.7)	5 (1.2)	0.182
Endocrine disorder	5 (1.0)	0 (0.0)	5 (1.2)	0.548
Laboratory manifestation				
Total leucocyte count (x10^3^cells/mm3), median (IQR) (n = 354)	7.8 (5.3–11.1)	10.5 (7.1–13.0)	7.6 (5.2–10.7)	0.003
Platelet (x10^3^cells/mm^3^), median (IQR) (n = 354)	221.9 ± 97.4	187.6 ± 97.9	226.3 ± 96.6	0.018
Albumin (g/dl), mean ± SD (n = 350)	3.99 ± 0.70	3.23 ± 0.72	4.09 ± 0.63	<0.001
AST (IU/L), median (IQR) (n = 350)	18.0 (12.0–31.0)	25.0 (14.1–52.5)	18.0 (12.0–29.0)	0.028
Total bilirubin (mg/dl), median (IQR) (n = 350)	0.5 (0.3–0.7)	0.5 (0.3–0.7)	0.5 (0.3–0.6)	0.492
Creatinine (mg/dl), median (IQR) (n = 350)	0.80 (0.60–1.30)	1.5 (1.1–2.2)	0.8 (0.6–1.13)	<0.001
LDH (IU/L), median (IQR) (n = 316)	228.1 (182.0–325.0)	396.0 (263.0–686.0)	219.0 (178.0–307.5)	<0.001
CRP (mg/l), median (IQR) (n = 346)	43.4 ± 64.5	95.2 ± 87.0	36.6 ± 57.7	<0.001
Lactate, median (IQR) (n = 320)	1.2 (0.9–1.8)	2.4 (1.1–13.7)	1.2 (0.8–1.7)	<0.001

*Note*: Data are n (%) unless otherwise stated.

AST, aspartate aminotransferase; CRP, C-reactive protein; ED, emergency department; IQR, interquartile range; LDH, lactate dehydrogenase; SD, standard deviation.

### Comparison of characteristics between non-survivors and survivors

Bivariate analyses revealed significant differences in clinical and laboratory characteristics between patients who survived compared to those who did not (Table 1). The non-survivor group was much older (mean, 71.9 years vs. 56.4 years, *P* < 0.001) and had a lower rate of full vaccination (53.7% vs. 68.2%, *P* = 0.034). Underlying chronic lung diseases (9.3% vs. 2.4%, *P* = 0.019), liver cirrhosis (7.4% vs. 1.9%, *P* = 0.036), chronic renal diseases (22.2% vs. 10.4%, *P* = 0.001), and AKI at presentation (11.1% vs. 2.4%, *P* = 0.005) significantly affected 30-day mortality. In terms of serologic testing, the non-survivor group exhibited remarkably higher leukocyte counts, AST, creatinine, LDH, CRP, and lactate levels. In addition, the non-survivor group displayed significantly lower levels of albumin and platelet counts.

### Predictors of mortality of COVID-19 patients presenting to the ED

Multivariate Cox proportional hazards model revealed that old age (hazard ratio [HR], 1.042; 95% confidence interval [CI], 1.012–1.074; *P* = 0.007), AKI at presentation (HR, 5.078; 95% CI, 1.856–13.894; *P* = 0.002), total leukocyte counts (HR, 1.080; 95% CI, 1.026–1.138; *P* = 0.004), platelet counts (HR, 0.993; 95% CI, 0.988–0.998; *P* = 0.007), albumin levels (HR, 0.224; 95% CI, 0.121–0.412; *P* < 0.001), and LDH levels (HR, 1.002; 95% CI, 1.001–1.002; *P* < 0.001) were the independent predictors of mortality. ROC curve analyses revealed that albumin levels had the highest discriminative ability (AUC, 0.860; 95% CI, 0.796–0.875) for mortality compared to LDH (AUC, 0.664; 95% CI, 0.613–0.713), leukocyte (AUC, 0.648; 95% CI, 0.596–0.697) and platelet counts (AUC, 0.644; 95% CI, 0.592–0.693) (Supplementary Figure S1). Significant differences were observed between the AUC of albumin levels and those of other markers such as LDH (*P* = 0.003), platelet counts (*P* < 0.001), and total leukocyte counts (*P* < 0.001), while no significant differences were found in other pairwise comparison of ROC curves (*P* > 0.05). An optimal albumin threshold of 3.7 g/dL predicted mortality with 82.5% (95% CI, 67.2–92.7) sensitivity and 77.4% (95% CI, 72.8–81.5) specificity (Supplementary Figure S2).

## Discussion

This study provides critical insights into the diverse clinical presentations and potential prognostic markers in adult COVID-19 patients admitted to the ED. Beyond the well-documented respiratory symptoms, our findings highlight a wide spectrum of extrapulmonary manifestations observed in these patients. The observed mortality rate of 11.3% in this cohort further emphasizes the gravity of the disease, particularly among patients presenting to the ED. Our results also demonstrated that serum albumin level may serve as a novel and simple early biomarker to identify COVID-19 patients at high risk for mortality.

We found that 46.4% of COVID-19 patients exhibited extrapulmonary manifestations, most commonly gastrointestinal, neurological, and cardiac issues. This finding aligns with emerging data that SARS-CoV-2 can induce multi-organ dysfunction [1, 2]. Extrapulmonary presentations in COVID-19 have garnered increasing attention, and our study aligns with previous research indicating that COVID-19 can initially manifest with non-respiratory symptoms [1, 2]. A previous cohort study involving 147 emergency medical services for COVID-19 in the United States found that 29.3% of patients did not present with respiratory symptoms and instead had a variety of symptoms, including altered mental status, chest pain, weakness, and pain or minor injury, often resulting from a fall [6]. Our study results are consistent with those of previous studies documenting the diverse clinical presentations associated with COVID-19 and highlight the importance of considering extrapulmonary symptoms in the diagnosis and management of patients.

The overall mortality rate was 11.3% in our cohort. Regarding predictors of mortality, our analysis identified that old age, AKI at presentation, increased total leukocyte counts, low platelet counts, decreased albumin levels, and increased LDH levels were significant factors associated with 30-day mortality in patients with COVID-19. Old age, underlying comorbidities like diabetes, chronic lung disease, cardiovascular disease, hypertension, cancer, obesity, AKI, and increased D-dimer are well-established risk factors for mortality in COVID-19 patients [7]. In addition, inflammatory biomarkers such as procalcitonin, cardiac markers, WBC, lactate, creatinine, D-dimer, LDH, CRP, AST, interleukin 6 (IL-6), BNP, blood urea nitrogen, creatine kinase, bilirubin, and erythrocyte sedimentation rate levels have been found to be increased in severe/fatal COVID-19 and predictive of poor prognosis [4, 5]. Other lab parameters such as lymphopenia, coagulation abnormalities, and decreased levels of albumin have also predicted COVID-19 mortality. The objective of our research was to identify a simple and readily obtainable biomarker measured early during hospitalization that could predict mortality. Therefore, we additionally conducted ROC analysis to assess the diagnostic sensitivity of independent predictors for 30-day mortality in patients with COVID-19. Total leukocyte counts, low platelet counts, albumin levels, and LDH levels were included in the ROC analysis. Notably, serum albumin levels exhibited a remarkably high AUC in ROC analysis, indicating its strong discriminatory power in predicting mortality. This outperformed other markers such as LDH levels, platelet counts, and total leukocyte counts. The diagnostic sensitivity of albumin levels for predicting mortality in patients with COVID-19 at a cut-off value of 3.7 g/dl was 82.5% (95% CI, 67.2–92.7), and the specificity was 77.4% (95% CI, 72.8–81.5). The serum albumin level serves as a frequently evaluated biomarker in hospitalized patients. Diminished levels of serum albumin are evident across various disease states and are linked to heightened in-hospital mortality and prolonged duration of hospitalization [8, 9]. They have been posited as a dependable prognostic indicator for outcomes in critically ill patients affected by infectious diseases [8, 9]. Malnutrition, diminished hepatic synthesis, renal losses, and inflammation are identified as primary contributors to hypoalbuminemia; nevertheless, in critically ill patients, the chief mechanism leading to a reduction in serum albumin is primarily attributed to increased capillary permeability and redistribution from the plasma to the interstitium [10]. In our investigation, while the correlation between albumin levels and the severity of the underlying condition or the acuteness of illness was not definitively established, our study underscores the potential value of albumin levels as a conveniently accessible marker for risk stratification in individuals with COVID-19.

Although our study offers valuable information on the characteristics and outcomes of COVID-19 patients presenting to the ED, it is essential to recognize and consider several limitations when interpreting the results. First, this study was conducted in a single centre, which could restrict the generalizability of the findings to other healthcare settings or diverse populations. Regional variations in patient demographics, vaccination rates, and healthcare resources could have affected the observed results. Therefore, caution should be exercised when extrapolating these findings to broader populations. Second, we did not incorporate other markers that have been associated with the outcomes of infectious diseases including cytokines (e.g., IL-6). Third, the study focused on patients who presented to the ED, which may have introduced a selection bias. Patients with mild symptoms who did not seek emergency care or were managed in outpatient settings were excluded from the analysis. Consequently, the study population may be skewed toward individuals with more severe diseases or specific clinical presentations, potentially influencing the observed results. Fourth, the sample size of the study, particularly within certain subgroups (e.g., underlying diseases), may have limited statistical power to detect significant differences or associations. A larger sample size would enhance the robustness and reliability of the findings, allowing for more comprehensive subgroup analyses and adjustments for potential confounders. Fifth, we conducted correlation analyses between age, liver cirrhosis, and serum albumin levels prior to proceeding with the multivariate analysis model and found significant associations. However, the variation inflation factor values of age and liver cirrhosis were 1.452 and 1.028, respectively. Consequently, we can conclude that multicollinearity was not present in our model. Nonetheless, there is the potential for residual confounding arising from factors, which may influence both albumin levels and mortality risk. Lastly, the optimal albumin threshold of >3.5 g/dL for predicting higher 30-day mortality was higher than the typically referenced normal range. A potential explanation is that even mildly decreased albumin levels may signify increased vulnerability and mortality risk when precipitated by an acute illness requiring hospitalization, especially in an elderly population. However, this higher than expected threshold is an interesting finding that warrants further study to better understand if it represents a population-specific normal range or highlights albumin’s role as a biosensor of overall health and resiliency.

In conclusion, our study sheds light on the diverse clinical presentations and critical prognostic markers in COVID-19 patients presenting to the ED. Extrapulmonary manifestations were prevalent, emphasizing the need for a comprehensive approach to assessment and monitoring. Old age, AKI at presentation, increased total leukocyte counts, low platelet counts, decreased albumin levels, and increased LDH levels emerged as pivotal factors influencing mortality, with albumin exhibiting particularly strong predictive power. Attending physicians should maintain a vigilant approach to recognize the potential extrapulmonary manifestations of COVID-19 patients in the ED, and serum albumin levels could help enhance situational awareness and rational resource allotment amidst the dynamically evolving COVID-19 situation.

## Supporting information

Park et al. supplementary material 1 Park et al. supplementary material

Park et al. supplementary material 2 Park et al. supplementary material

Park et al. supplementary material 3 Park et al. supplementary material

## Data Availability

The data are available from the corresponding author upon reasonable request.

## Supplementary material

The supplementary material for this article can be found at http://doi.org/10.1017/S0950268824000633.

## References

[1] Gupta A, et al. (2020) Extrapulmonary manifestations of COVID-19. Nature Medicine 26, 1017–1032. 10.1038/s41591-020-0968-3 PMC11972613 32651579

[2] Lai CC, et al. (2020) Extra-respiratory manifestations of COVID-19. International Journal of Antimicrobial Agents 56, 106024. 32450197 10.1016/j.ijantimicag.2020.106024 PMC7243791

[3] Leblanc J, et al; DEPIST-COVID group and FHU IMPEC (Improving Emergency Care) group (2023) Intensified screening for SARS-CoV-2 in 18 emergency departments in the Paris metropolitan area, France (DEPIST-COVID): A cluster-randomized, two-period, crossover trial. PLoS Medicine 20, e1004317. 38060611 10.1371/journal.pmed.1004317 PMC10735176

[4] Izcovich A, et al. (2020) Prognostic factors for severity and mortality in patients infected with COVID-19: A systematic review. PLoS One 15, e0241955. 33201896 10.1371/journal.pone.0241955 PMC7671522

[5] Battaglini D, et al. (2022) Laboratory biomarkers for diagnosis and prognosis in COVID-19. Frontiers in Immunology 13, 857573. 35572561 10.3389/fimmu.2022.857573 PMC9091347

[6] Yang BY, et al. (2020) Clinical characteristics of patients with coronavirus disease 2019 (COVID-19) receiving emergency medical services in King County, Washington. JAMA Network Open 3, e2014549. 32639570 10.1001/jamanetworkopen.2020.14549 PMC7344378

[7] Dessie ZG and Zewotir T. (2021) Mortality-related risk factors of COVID-19: a systematic review and meta-analysis of 42 studies and 423,117 patients. BMC Infectious Diseases 21, 855. 34418980 10.1186/s12879-021-06536-3 PMC8380115

[8] Artero A, et al. (2010) Prognostic factors of mortality in patients with community-acquired bloodstream infection with severe sepsis and septic shock. Journal of Critical Care 25, 2762–2781. 10.1016/j.jcrc.2009.12.004 20149587

[9] Wi YM, Kim JM and Peck KR. (2014) Serum albumin level as a predictor of intensive respiratory or vasopressor support in influenza a (H1N1) virus infection. International Journal of Clinical Practice 68, 222–229. 24372959 10.1111/ijcp.12249

[10] Foley EF, et al. (1990) Albumin supplementation in the critically ill. A prospective, randomized trial. Archives of Surgery 125, 739–742. 2111981 10.1001/archsurg.1990.01410180063012

