# Supplementary figures and images for: Characteristics and biomarkers associated with mortality in COVID-19 patients presenting to the emergency department
Source: Epidemiol Infect. 2024 Apr 19;152:e76. doi: 10.1017/S0950268824000633 (PMC11094378; doi:10.1017/S0950268824000633)

## Slide 1
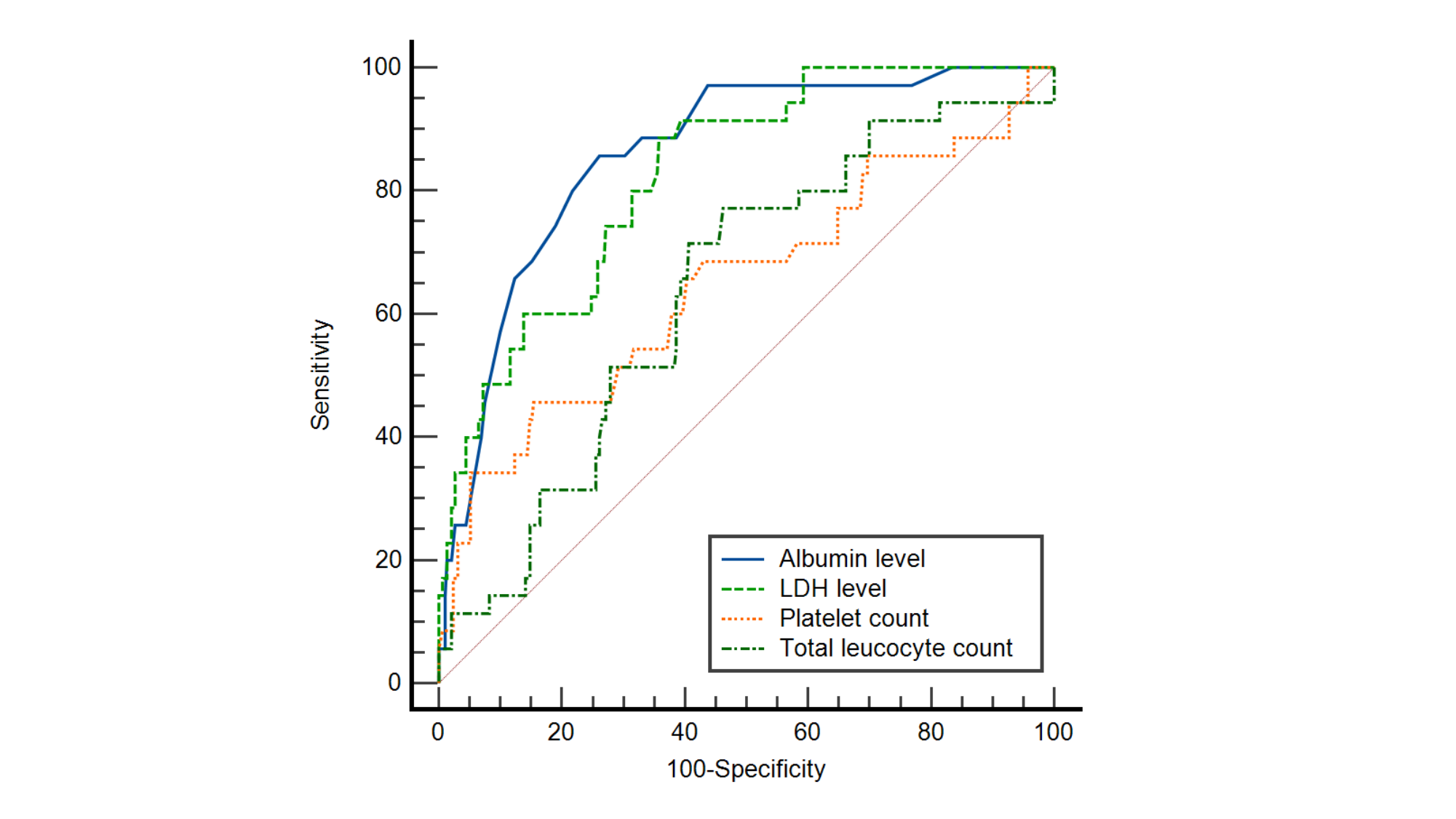

Supplement: Park et al. supplementary material 1 — Park et al. supplementary material [file S0950268824000633sup001.pptx]

## Slide 1
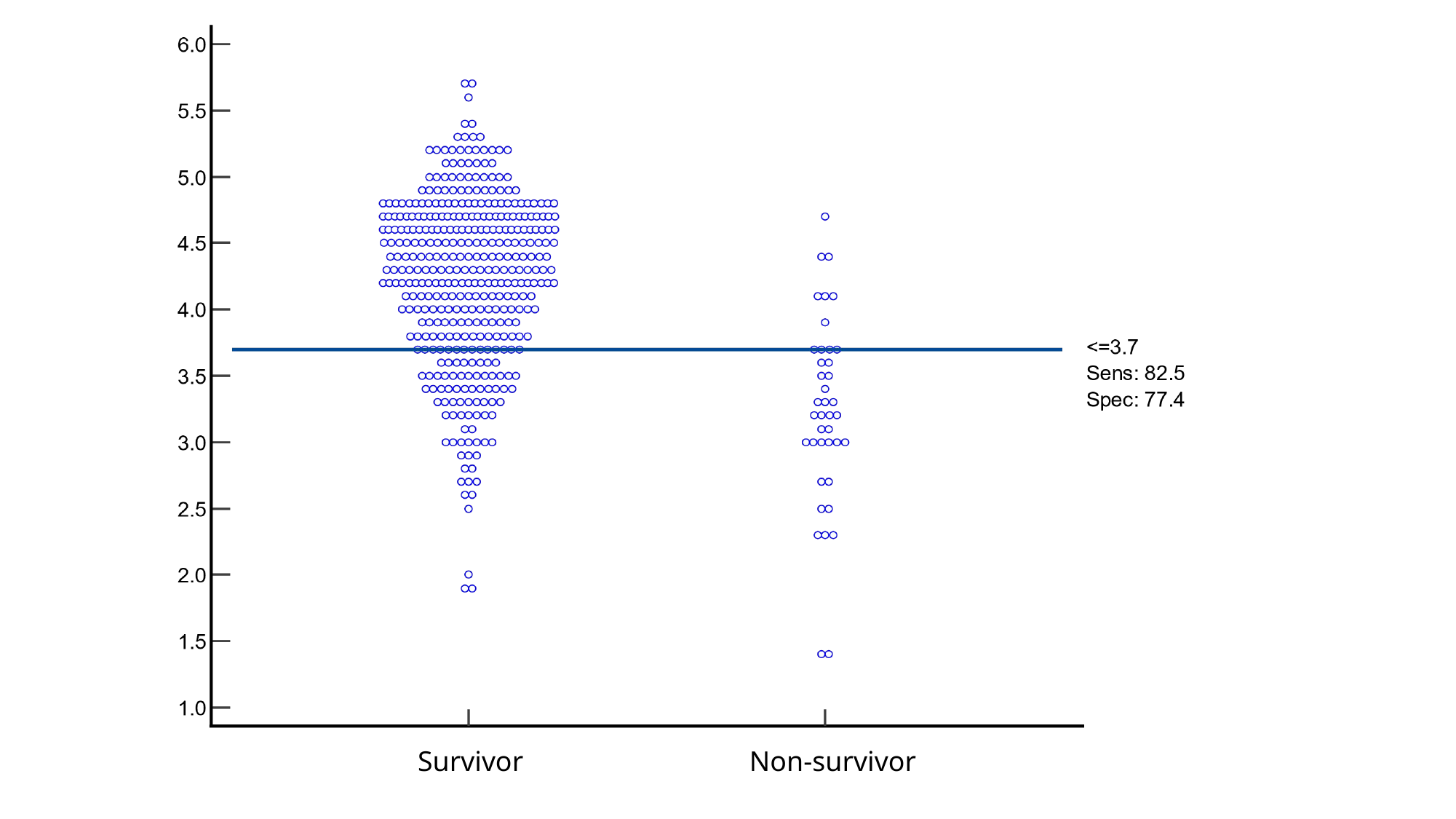

Survivor
Non-survivor

Supplement: Park et al. supplementary material 2 — Park et al. supplementary material [file S0950268824000633sup002.pptx]
