# Supplementary material for: Characteristics and biomarkers associated with mortality in COVID-19 patients presenting to the emergency department
Source: Epidemiol Infect. 2024 Apr 19;152:e76. doi: 10.1017/S0950268824000633 (PMC11094378; doi:10.1017/S0950268824000633)
Supplement: Park et al. supplementary material 3 — Park et al. supplementary material [file S0950268824000633sup003.docx]

**Supplement Figure Legends**

**Supplement Fig 1.** ROC curves for albumin, lactate dehydrogenase (LDH), platelet, and total leucocyte count for predicting of mortality in patients with COVID-19 presenting to the emergency room (n=359). The AUC was 0.860 (95% CI 0.796–0.875) for albumin, 0.664 (95% CI 0.613–0.713) for lactate, 0.648 (95% CI 0.596–0.697) for total leucocyte count, and 0.644 (95% CI 0.592–0.693) for platelet count.

**Supplement Fig 2.** The diagnostic sensitivity and specificity of albumin levels at a cut-off value of 3.7 g/dl for predicting of mortality in patients with COVID-19 presenting to the emergency room.
